# Exploring the landscape of livestock ‘Facts’

**DOI:** 10.1016/j.gfs.2019.100329

**Published:** 2020-06

**Authors:** G.R. Salmon, M. MacLeod, J.R. Claxton, U. Pica Ciamarra, T. Robinson, A. Duncan, A.R. Peters

**Affiliations:** a Supporting Evidence-Based Interventions (SEBI), The Royal (Dick) School of Veterinary Studies, The University of Edinburgh, Easter Bush Campus, Midlothian, EH25 9RG, UK; b Scotland's Rural College (SRUC), West Mains Road, Edinburgh, EH93JG, UK; c Institute of Biodiversity Animal Health & Comparative Medicine, Graham Kerr Building, University of Glasgow, Glasgow, G12 8QQ, UK; d Animal Production and Health Division, Food and Agriculture Organization of the United Nations (FAO), Viale delle Terme di Caracalla, 00153, Rome, Italy; e Global Academy of Agriculture and Food Security, University of Edinburgh, Edinburgh, UK; f International Livestock Research Institute, PO Box 5689, Addis Ababa, Ethiopia

**Keywords:** Livestock, Communication, Facts, Evidence

## Abstract

The role of livestock in supporting human well-being is contentious, with different perceptions leading to polarised opinions. There is increasing concern about the health and environmental impacts of a high rate of consumption of livestock products in high-income countries. These concerns are heightened by an increase in consumption in middle-income countries. On the other hand, livestock support the livelihoods of many people, particularly in low income countries. The benefits of livestock for poor livestock keepers are multiple, including the important role livestock play in supporting crop production in mixed systems, in supplying nutrients and income, and in fulfilling cultural roles. In addition livestock can provide resilience against economic and climate shocks. In view of these apparent positive and negative impacts, the role of livestock in human wellbeing is highly contested, with arguments ‘for’ or ‘against’ sometimes distorted by vested interests or misinterpretation of evidence. The Livestock Fact Check project, undertaken by the Livestock Data for Decisions community of practice, has investigated several ideas concerning livestock commonly taken as ‘fact’. By exploring the provenance of these ‘facts’ we highlight their importance and the risks of both misinterpreting them or using them out of context. Despite the diversity of the livestock sector resulting in equally diverse viewpoints, the project calls for participants in the livestock discourse to adopt a nuanced appreciation of global livestock systems. Judgement of livestock's role in global sustainable diets should be based on clear and well-interpreted information.

## Highlights

• Livestock farming is a contentious and polarised subject in sustainable diet discourse.
• Livestock facts are used to support rationales from a variety of positions.
• Many ‘facts’ are used without acknowledgement of their provenance or without suitable interpretation.
• Knowing what is uncertain or variable is important for fair interpretation, dialogue and rational decisions.
• Transparency of evidence related to livestock is the ambition of the Livestock Data for Decisions community of practice.

## 1 Introduction

**Fig. 1:**
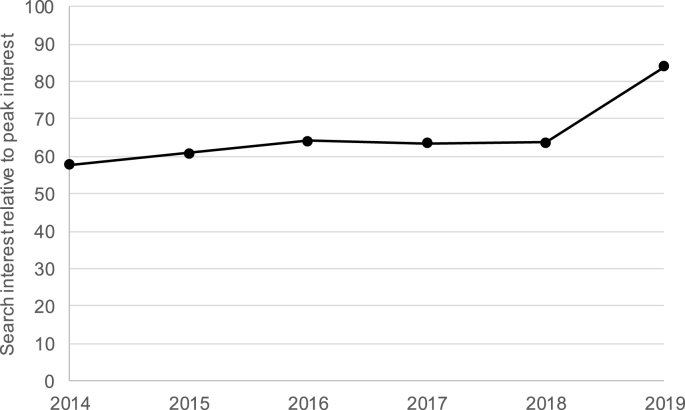
Global trend in the use of Google search term ‘Livestock’. The y-axis numbers 0–100 represent search interest relative to the highest point on the 2014–2019 chart. A value of 100 is the peak popularity for the term (which occurred in March 2019). A value of 50 means that the term is half as popular. Data from Google trends (Google, 2019).

Livestock production makes a significant contribution to human existence; recent estimates suggest that the global biomass of livestock is twice that of human populations (Bar-On et al., 2018). Since livestock were first domesticated, some 10,000 years ago, their production has played a significant role in the development of civilisation (FAO, 2007). Recent decades have seen many programmes and investments to support the development of livestock production. These include significant programs in developed countries to modernise breeds (García-Ruiz et al., 2016), the eradication of rinderpest disease globally (Roeder et al., 2013) and increases in dairy production in India through Operation Flood (Cunningham, 2009). Simultaneously, there has been a growing awareness of the negative consequences of livestock production. These include environmental damage (Steinfeld et al., 2006), poor animal welfare (Robbins et al., 2016), human illness due to zoonotic diseases (Gebreyes et al., 2014) and ill health due to a high consumption of livestock products (Godfray et al., 2018), as well as the rise in antibiotic resistance (FAO, 2016a). As such livestock sector discourse is increasingly of interest to the wider population (Stevens et al., 2018) (Fig. 1), and remains a contentious topic (Busch and Spiller, 2018).

In livestock science, as with any scientific discipline, there are a minority of experts amongst a majority population of non-experts; both access evidence relevant to their situations and choices (Bromme and Goldman, 2014). It is important that such evidence is well intrepreted. The impact of misinterpretation on society, in a “post-truth” era, is explored by Lewandowsky et al. (2017). An example impact is the decline in childhood measles, mumps and rubella (MMR) vaccination following communication of a single flawed study in 1998, and the more recent rise in a broader anti-vaccination movement (Hussain et al., 2018). Genetically modified organism (GMO) food sources are an example within agriculture. Despite scientific consensus suggesting that there is no greater risk to the environment or consumer health in consuming GMO food than conventional food, GMO food is not globally accepted or utilised (Yang and Chen, 2016; Scott et al., 2018). While some may oppose GMO with concerns that large companies may gain power and influence over producers (Dibden et al., 2013); the debate is often impervious to science and instead guided by emotional and moralising rhetoric (Hielscher et al., 2016; Scott et al., 2018).

Perceptions and opinions of the global livestock sector are not only diverse but are also becoming increasingly polarised (Busch and Spiller, 2018). For instance, based on evidence concerning negative aspects of livestock, the proportion of the population in high-income countries following vegan diets is rapidly increasing (Gill et al., 2018). With the same evidence, some commentators call for a livestock-free world (Monbiot, 2017). Such a stance ignores the role livestock production plays in supporting the livelihoods of large populations in low- and middle-income countries (LMICs) (Herrero et al., 2013a). By improving communication and interpretation of livestock evidence, including both negative and positive impacts of the livestock sector, today's polarised views might be replaced by a more constructive dialogue concerning livestock's role in humanity's future.

Livestock Data for Decisions (LD4D) is a community of practice, with members from academia, NGOs, donor agencies and industry. LD4D's aim is to ‘drive informed livestock decision-making through better use of existing data and analyses’ (LD4D, 2019). This multi-disciplinary community, seeing that livestock sector ‘facts’ are regularly communicated without a full understanding of their provenance and validity, initiated a Livestock Fact Check project to identify and investigate popular ‘facts’ relating to important aspects of livestock systems. This project encouraged dialogue to help ensure livestock production discussions and decisions are well informed, facts appropriately interpreted, and gaps in knowledge and assumptions recognised. This paper summarises the findings of the project's investigations.

## 2 Investigating popular livestock ‘facts’

The livestock ‘facts’ investigated by LD4D refer both to quantitative evidence, such as a specific percentage or quantity, and to qualitative evidence, such as a broad perception or understanding. The ’facts' to be investigated were identified by a multidisciplinary working group representing the broader multi-discplinary LD4D community of practice. To help promote constructive community dialogue, efforts were made to select ‘facts’ that represented key aspects of livestock production (e.g. economy, environment, livelihoods and health). While the methodologies used to investigate each ‘fact’ were variable, efforts were made in every case to identify the origins of the ‘facts’ through interrogations of the relevant literature. The ‘facts’ investigated are summarised in the following sections.

### 2.1 Livestock supporting the livelihoods of poor people

Publications on livestock development have regularly quoted ‘one billion’ as being the global number of poor people supported by livestock, typically to demonstrate the importance of livestock to humanity. In some instances this number is not given a reference (e.g. FAO, 2009); more commonly, a secondary source is cited (e.g. Thorne and Conroy, 2017). Tracing back through publications referencing ‘one billion’ suggests that the original source is a study published in 1999 by Livestock in Development (LID). The UK Department for International Development (DFID) funded that study to examine ‘*the case for investment in the livestock sector as a basis for reducing rural poverty*’ (LID, 1999). LID calculated that the global number of poor livestock-keepers was 987 million (rounded up to one billion). For this calculation, LID used a global livestock-keeper agro-ecological distribution reported by Seré and Steinfeld (1996) and poverty statistics from the United Nations Development Programme (1997). In turn, the livestock-keeper distribution was based on 1991–1993 data from AGROSTAT (now FAOSTAT) and the poverty statistics used a composite poverty measure cited as “*correspondence on the Gini coefficient*” from the World Resources Institute in 1996 (no further information was available relating to this correspondence). Consequently, the regularly quoted ‘one billion’ statistic is based on a calculation using previous publications and statistics, all more than 20 years old. Between 1999 and 2017, the global human population has grown from 6.1 to 7.6 billion (FAOSTAT, 2019); growth is concentrated in LMICs and unlikely to slow in the foreseeable future (Gerland et al., 2014). Globally, there is net rural to urban migration, with a higher proportion of the population living in urban centres than in rural locations since 2007 (FAO, 2018d; FAOSTAT, 2019). Recent economic modelling suggests that for those remaining rural populations, particularly farmers, poverty is limiting, with economic growth suggested to be low or even negative (Castañeda et al., 2018; Laborde Debucquet and Martin, 2018). Additionally, the global definition of ‘poor’ has changed; the World Bank's one-dollar-a-day indicator, tracking ‘*the share of individuals that have to live on less than an absolute minimum*’, was adjusted three times between 1999 and 2015 (Klasen et al., 2016). Today, the number of people living in ‘extreme poverty’, below the USD-1.9-a-day poverty line, is estimated to be 731 million (The World Bank, 2019), which invalidates the ‘one billion’ poor livestock keepers statistic. There are more recent calculations to suggest livestock's significance in supporting the global poor (Herrero et al., 2009; Robinson et al., 2011); however, variation in methods and applied data mean trends cannot be easily assessed. Accuracy may not be of high importance in certain applications of this ‘fact’. For instance, the order of magnitude could be enough to communicate that a ‘large number’ of poor people depend on livestock, and therefore livestock should not be ignored in future food security efforts (FAO, 2018c; World Economic Forum, 2019). Whereas to inform specific investment and development actions (and monitor subsequent impact) it would be useful to understand trends (requiring increased accuracy and a consistent methodology) in the importance of livestock at both local and global scales. Interestingly, more recent publications demonstrate the need to give livestock development consideration by drawing attention to the importance of livestock to stakeholders across associated value chains, not just poor livestock keepers (World Economic Forum, 2019).

### 2.2 Livestock multifunctionality

Global livestock production discussions tend to focus on issues of importance to high-income countries and on just a few dimensions of livestock systems, notably their environmental impacts and the harm to human health that can be caused by high consumption of animal-source foods and zoonotic diseases. Whilst the myriad of functions of livestock in LMICs are commonly referenced in livestock development communications (FAO, 2012; Herrero et al., 2013a), they tend to be overlooked or downplayed in global level discourse (for example consider Godfray et al. (2018) or Willett et al. (2019)). However, the roles of livestock in supporting crop production with draught power and manure; providing a valuable use for crop residues and other by-products; providing high-quality nutrition, a regular income, insurance and savings; as well as cultural and social roles, should not be ignored (Moll et al., 2007; FAO, 2012). The evidence for these functions is extensive and comes from various methodologies, including ethnographic observations, participatory rural appraisals, interviews, focus groups and surveys, as well as literature reviews and modelling exercises (Freeman et al., 2008; Ejlertsen et al., 2013; Herrero et al., 2013a; Marshall, 2014; Quinlan et al., 2016).

**Fig. 2:**
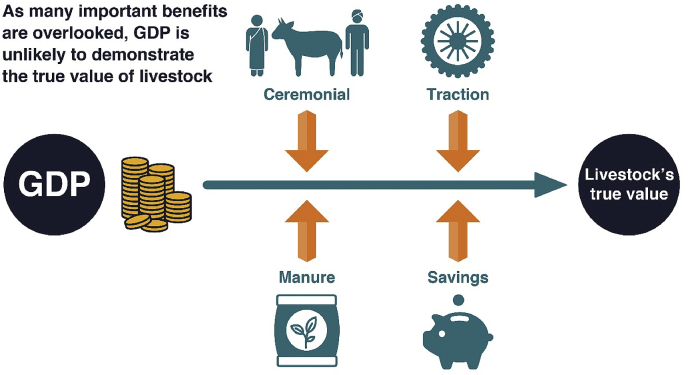
The Livestock Fact Check project created several infographics to improve the communication of key messages. The above example demonstrates the importance of considering all the various benefits of livestock to appreciate a true value. Significantly, this infographic does not include all the costs (internal and external) for a true net value.

Recognising the different ways to value livestock adds significant complexity to already complex and impassioned discussions and decisions. For instance, data from household surveys can be used to measure monetary income from livestock, but it is questionable as to whether this metric is a fair evaluation of livestock's contributions to household wellbeing (for instance consider all the benefits identified in Fig. 2). Estimations of the extent of poverty amongst livestock keepers could potentially be reduced if the value of their livestock income is increased to take into account the many non-tradeable outputs and services farm animals provide. Another example is the allocation of greenhouse gas emissions to different livestock products, with the objective of comparing their environmental impacts. Traditionally, these estimates are based on a standard unit such as kg of emissions per kg of protein (Gerber et al., 2013). However, as livestock in LMICs have value beyond the production of protein, it has been suggested that estimates of emission intensities should take account of these additional values (Weiler et al., 2014). Recognising multiple functions also complicates efforts to enhance the sustainability of livestock systems, with trade-offs between functions likely to exist (Salmon et al., 2018). For instance, if the objective in LMICs were to increase food production, there would likely be efforts to shift from ‘low-input, low-output’ to ‘high-input, high-output’ production systems, which will in turn impact the other functions livestock provide society. As a result, evidence suggests that such a shift does not necessarily lead to increased income or improved nutrition for livestock-keepers and can reduce resilience (Salmon et al., 2018). Conversely, ‘high-input, high-output’ production systems could be more efficient at providing affordably priced animal-source food to non-livestock keepers, thereby improving food security and nutrition at a national scale (FAO, 2009). A good understanding of livestock multi-functionality is mandatory to ensure future transitions are positive at multiple dimensions.

### 2.3 Livestock's contribution to the economy

**Fig. 3:**
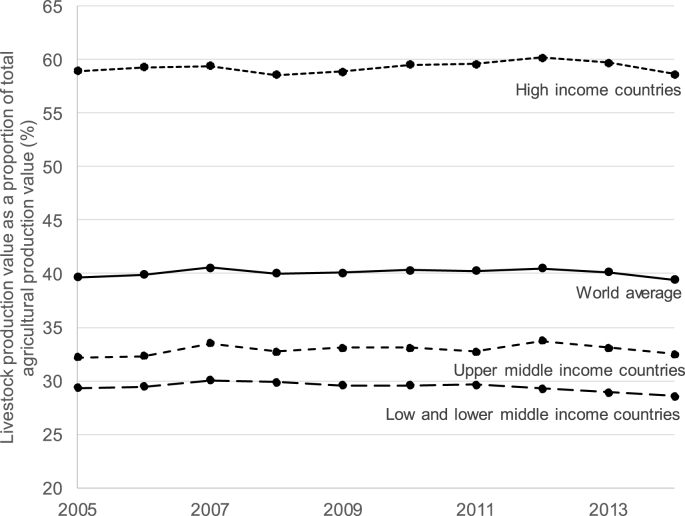
The contribution of livestock gross production value to total agricultural gross production value (used to suggest livestock contribution to total agricultural GDP). Countries grouped as per World Bank defined income groups for 2010, data source FAOSTAT (FAO, 2012; FAOSTAT, 2019).

To demonstrate the importance of livestock, it is regularly reported that globally livestock production accounts for 40% of total agricultural gross domestic product (GDP) (The World Bank, 2009; FAO, 2018a). This 40% contribution statistic has become current and is often expressed without question or reference to an original calculation. Investigation suggests that the 40% figure originates from calculations made for the publication ‘Livestock's Long Shadow’, with the objective of demonstrating the political, social and economic importance of livestock (Steinfeld et al., 2006). The calculation was based on FAOSTAT data on the value of agricultural production from 2005, though it is unclear whether the figure referred to gross or net values. A recalculation, using the FAOSTAT gross value of production data from 2005 to 2014, suggests there has not been any significant change in the 40% contribution of global livestock production to total agriculture (Fig. 3). Yet it is evident that a global figure obscures significant regional variation. Notably, on average livestock production in LMICs appears to contribute less than 40% to total agricultural production value and has remained relatively stable between 2005 and 2014. Again, there is variation behind a LMIC average, suggesting that for some LMICs (for instance Somalia) livestock are of particular importance to economy (contributing over 80% of total agricultural production value) and for others (for instance Côte d'Ivoire) the economy relies heavily on crop production (Addison et al., 2016), with livestock contributing less than 10% of total agricultural production value (based on 2014 FAOSTAT data). FAOSTAT data also suggests that as a country's wealth increases the share of livestock GDP of total agriculture GDP grows, but agriculture's contribution to total national GDP decreases. Evidently, there is a risk in using broad scale averages for livestock communications, and that citing the 40% figure to demonstrate increased need to invest in livestock for development can be misleading. It can also be questioned if the value of production is a fair measure of the value of livestock for society in LMICs, as livestock have values beyond production (as mentioned in the previous section) that are unlikely to be captured in national accounting (FAO, 2012; Morgan and Pica-Ciamarra, 2012). For instance, where efforts have been made to include the value of draught power and the provision of manure, the total contribution of livestock to GDP more than doubled in Ethiopia, where cattle draught power is significant (Behnke, 2010; IGAD, 2013). Other values, such as the ceremonial and status value of livestock, are even harder to quantify, but their significance in LMICs should not be ignored (Herrero et al., 2013a). In addition, the possible negative externalities generated by livestock production, such as environmental degradation, risks to human health through zoonotic diseases, and livestock-driven antimicrobial resistance, are not captured in the value of production or more broadly in GDP calculations (Van Den Bergh, 2009).

### 2.4 Livestock and disease

Zoonotic diseases are those transmitted between humans and animals. The control of livestock disease to benefit humans is encouraged using ‘facts’ such as ‘60% of human pathogens are zoonotic, and 75% of emerged human epidemics are of animal origin’ (Karesh et al., 2012; Gebreyes et al., 2014; Cunningham et al., 2017). These figures originated in 2001, when Taylor et al. reviewed 39 publications (dated from 1975 to 2000) relating to human pathogens. Eight hundred and sixty-eight (≈60%) of the 1415 identified species of infectious agents causing human disease were zoonotic, including 132 (75%) of 175 pathogens associated with human emerging infectious diseases (EID) (Taylor et al., 2001). An update of the 2001 database in 2005 reported similar figures (Woolhouse and Gowtage-Sequeria, 2005). A further review in 2008, highlighted the additional importance of wildlife populations; 747 references (dated from 1940 to 2004) concerning 335 distinct EID events suggested that 60% were zoonotic, but of these 72% had an original wildlife origin (Jones et al., 2008). Livestock play a significant role in transmitting diseases from wildlife to human populations; which also gives livestock a role as sentinels for early detection of costly zoonotic pandemics amongst human populations (Ma et al., 2009; The World Bank, 2012). The impacts of zoonoses are broad and significant (The World Bank, 2012; FAO, 2018b), therefore a good understanding of their origin and transmission is vital to inform decision makers and allow effective allocation of resources. The ‘One Health’ paradigm is growing in recognition of this (promoting collaboration between human and veterinary healthcare and their interaction with environmental issues) (Cunningham et al., 2017).

In addition to directly harming human health, livestock disease also reduces livestock production itself (for instance, through animal deaths and illness, reduced growth rates, and decreases in milk or egg yields). Those advocating investment to improve animal health commonly state that ‘*one quarter of the animals owned by poor livestock keepers die from preventable and treatable diseases*’ (New Scientist, 2008; BMGF, 2012). This ‘fact’ is regularly stated as common knowledge, with no accompanying citation. It is likely to be a derived ‘average’ from various mortality risks presented in a systematic review (of over 400 studies) for ruminant production systems in sub-Saharan Africa (Otte and Chilonda, 2002). Firstly, Otte and Chilonda (2002) did not claim that preventing the mortality they presented was an attainable target. Secondly, reporting a high level average figure masks important variations, which could assist in understanding the causes of and opportunities to prevent livestock mortality. Quantifying the primary impact of livestock diseases is notoriously challenging (Rushton et al., 2018). Initially there is a reliance on effective farm level recognition and reporting (Perry and Grace, 2009). Then, despite the mechanism by which identified diseases limit production being fairly well understood (FAO, 2010; OIE, 2018b; a), extent of burden is much harder to quantify and likely to vary (Perry and Grace, 2009). In some situations, case studies for particular diseases in specific production systems are extrapolated to estimate burdens for broader scenarios (Shaw et al., 2014; Knight-Jones et al., 2017). As a result, there is uncertainty around both the global impact of the livestock disease burden and the associated nuances. Both an increasing ability to detect and report disease (Rushton, 2017) and the recent launch of a Global Burden of Animal Disease (GBAD) programme to improve our understanding of the economic impact of animal disease (Rushton et al., 2018) are encouraging developments. Without being able to quantify primary impacts, the cost-effectiveness of interventions cannot be understood, limiting decision makers.

### 2.5 Livestock and environmental impact

The various negative environmental impacts of livestock production are well recognised; with global scale ‘facts’ relating to water consumption and pollution, land use changes, biodiversity losses and greenhouse gas emissions. These ‘facts’ are often used as headlines, with a mandate to reduce the consumption of livestock sourced foods in high income countries (e.g. Vidal, 2004; Mcwilliams, 2014; Cameron and Cameron, 2017; France-Presse, 2017; UN Environment, 2018). For instance it is often quoted that livestock supply chains are responsible for 14.5% of total human-induced greenhouse gas emissions; a global figure from life cycle assessment modelling (Gerber et al., 2013). Such global figures can be somewhat misleading when cited out of context, i.e. without the supporting analysis that explains the often considerable variation in environmental impact. The communication of such variation is an important part of enabling people to grasp opportunities to reduce environmental impact, particularly in scenarios where livestock production is necessary (i.e. where increased consumption of animal-sourced foods can benefit populations (Grace et al., 2018), or where livestock offer a unique source of livelihood (Turner et al., 2014)). In such scenarios, it is suggested that improving productivity offers mitigation opportunities; a message that relies on understanding variation in production systems (Gerber et al., 2011; Salmon et al., 2017). Equally, reporting such opportunities without supporting analysis can mislead through a lost understanding of what could be technically feasible and what is realistic when trade-offs in constrained scenarios are considered (Gerber et al., 2013; Herrero et al., 2016).

## 3 Discussion of cross-cutting issues

It is evident that many livestock ‘facts’ inherently relate to complex systems; which, to enable comprehension rely on systems modelling. There are important considerations to make when simplifying reality in this way. As discussed in previous sections, the quality, availability and resolution of livestock data will determine how closely modelled results illustrate reality. In addition modelled results are dependent on the scope and assumptions included, as well as the metrics selected to report results. The same production system could be investigated with different models reporting different metrics, producing results that are varied in their illustration of reality (Jones et al., 2016; Lynch, 2019). For instance estimates of the livestock sector's contribution to global GHG emissions have varied from 8 to 51% when modelling approaches varied (Herrero et al., 2011). If ‘facts’ produced by different models are to be compared, the methodologies, scope and assumptions must be well understood. Despite global assessment models' loss of resolution, they do encourage consistency and allow informed comparisons of production system scenarios (Herrero et al., 2013b; Macleod et al., 2017).

Science deals with uncertainty and variability and therefore cannot provide unqualified solutions to problems (Von Winterfeldt, 2013; Sinatra and Hofer, 2016). Global livestock production systems are characterised by uncertainty and variability, so the challenge to communicate livestock information appropriately is significant. The presentation of ‘facts’ by journalists wanting to summarise complex issues (Patterson, 2013), by politicians needing a rationale for a decision or position (Dietz, 2013), and by individuals arguing a particular point of view can mislead by suggesting more certainty than the data supports. In some of circumstances, the precision of ‘facts’ to a global order of magnitude may be appropriate. For instance knowing that the number of poor livestock-keepers is in the high hundreds of millions tells you livestock are important to poverty reduction and food security. Equally, in other circumstances or applications variability may be more informative. Knowing where the majority of poor livestock-keepers are located, or if there are trends in these populations could be more useful for strategic decisions by governments or development funders. Overlooking uncertainty and variability can be unhelpful. For instance, it is important that recipients of ‘facts’ about livestock's contribution to climate change are aware that these ‘facts’ largely come from modelling exercises with considerable recognised uncertainty. Likewise, the variability in emissions from different production systems is vital to acknowledge opportunities for more sustainable livestock production in the future. By recognising both uncertainty and variability, the users of livestock ‘facts’ can assess their fitness-for-purpose.

To enable an assessment of a ‘facts’ fitness-for-purpose (including the method of calculation or evidence and original purpose) one must understand its provenance; this relies on effective and appropriate referencing within communications. However, this Livestock Fact Check project highlighted that many livestock ‘facts’ are communicated without following this principle. Understandably, there are trade-offs between timely consumable scientific communication and traditional scientific principles. Nonetheless care should be taken to avoid assuming the accuracy of received information or interpretations before passing them on. For instance, a single interpretation (with potential flaws and caveats) can be magnified by repeated reference to secondary sources (making it appear that the interpretation was reached by multiple parties) rather than investigating the original single source (Rekdal, 2016).

Livestock data in general is challenging; data collection and reporting is often ad-hoc and inconsistent, with livestock often grouped in a broad category of agriculture (Pica-Ciamarra et al., 2014). Consequently, there are large knowledge gaps in the livestock science community. For instance, there is limited quantification of the production burdens of livestock disease, at both global and local scales. Without a good understanding of the primary impacts of livestock disease, we cannot understand or predict further secondary impacts such as lost income, reduced human nutrition, trade restrictions, or potential for environmental impact reduction (Perry and Grace, 2009; Shaw et al., 2014; FAO, 2016b; Skuce et al., 2016; Macleod et al., 2018; Mayberry et al., 2018).

To some extent, the ‘facts’ accepted by the broader population of non-experts will not be based on their quality or the quality of their communication. It is recognised that individuals are more likely to accept facts if they align with the values they hold or reinforce predispositions (Kahan et al., 2011; Kraft et al., 2015; Suhay and Druckman, 2015; Yeo et al., 2015; Bauer et al., 2016). For instance, a climate change skeptic is more likely to reject ideas about reducing meat consumption for global benefits (de Boer et al., 2013). Accordingly, it is not the intention of the current project to follow the classic ‘fact check’ approach and brand certain ‘facts’ as misinformation. Instead, by investigating the provenance of information now taken as ‘fact’, and highlighting both positive and negative aspects of livestock production, we encourage perceptions, discussions and decisions concerning livestock to be based on a broad landscape of fit-for-purpose ‘facts’.

## 4 Conclusion

This LD4D project investigated several popular conceptions of livestock production, regularly taken as ‘fact’, with the objective of ensuring discussions, decisions and rationales are well founded on appropriate research-based information. Several key messages became apparent during the project. Firstly and not uniquely for livestock science, to maintain clarity and context, communicators (in particular scientists) should make every effort to understand primary sources for information they wish to use (and cite them appropriately). It can then be clear how old, how accurate or how comprehensive the evidence is on which ‘facts’ and subsequent perceptions are based. The livestock community then needs to decide which ‘facts’ warrant recalculation or further investigation, presumably focusing on those that cause the most polarisation. To avoid misleading or inappropriate interpretations and use of ‘facts’, scientists need to remain clear and transparent about both uncertainties and variability in livestock production at both local and global scales. The application of scientific information outside the science community is to some extent uncontrollable; nevertheless, the livestock community must remain broadly objective and balanced in presenting information about global livestock production and both its future role in sustainable diets and impacts on broader sustainable development goals.

## Declaration of competing interest

Authors declare no conflict of interest.
